# Real-Time PCR Detection Patterns of Porcine Circovirus Type 2 (PCV2) in Polish Farms with Different Statuses of Vaccination against PCV2

**DOI:** 10.3390/v11121135

**Published:** 2019-12-08

**Authors:** Aleksandra Woźniak, Dagmara Miłek, Piotr Matyba, Tomasz Stadejek

**Affiliations:** 1 Department of Pathology and Veterinary Diagnostics, Institute of Veterinary Medicine, Warsaw University of Life Sciences—SGGW, Nowoursynowska 159C, 02-776 Warsaw, Poland; aleksandra_wozniak@sggw.pl (A.W.); dagmara_milek@sggw.pl (D.M.); 2 Department of Large Animal Diseases with Clinic, Institute of Veterinary Medicine, Warsaw University of Life Sciences—SGGW, Nowoursynowska 100, 02-797 Warsaw, Poland; piotr_matyba@sggw.pl

**Keywords:** porcine circovirus type 2, circulation, real-time PCR, serum, feces, oral fluid

## Abstract

Porcine circovirus type 2 (PCV2) is a globally spread pathogen controlled with generally highly efficacious vaccination protocols. In order to compare PCV2 detection profiles in farms with different vaccination statuses, serum (359) and fecal pools (351) and oral fluids (209) from four farms that do not vaccinate against PCV2 (NON-VAC) and from 22 farms that do vaccinate (VAC) were tested with quantitative real-time PCR. Additionally, nucleotide sequences of ORF2 of the virus were obtained from selected samples. Three genotypes, PCV2a, PCV2b, and PCV2d, were detected. Significant differences (*p* < 0.05) in PCV2 prevalence and quantities between the VAC and NON-VAC farms were evident. In five VAC farms, no viremia or shedding in feces was detected. On the other hand, in four VAC farms, the results were very similar to those from NON-VAC farms. No significant difference in PCV2 prevalence in oral fluids was observed between VAC and NON-VAC farms. An examination of viremia can be recommended for the detection of vaccination efficacy issues. The median of the PCV2 viral loads >6.0 log_10_ copies/mL in pooled sera from the vaccinated population should be considered a very strong indication that the vaccination protocol needs revision.

## 1. Introduction

Porcine circovirus type 2 (PCV2) is a member of the family *Circoviridae*, genus *Circovirus*, which contains small, non-enveloped viruses with a single-stranded circular DNA genome about 1.7–2.0 kb in size [1]. It is a primary causative agent of PCV2-associated diseases (PCVD), which include systemic illness, enteritis, pneumonia, and reproductive failure [2]. Porcine circovirus type 2 also causes subclinical disease without specific clinical signs, which is the most common PCV2 manifestation [2]. Thus, the virus has a significant economic impact on the swine industry [3,4]. Porcine circovirus type 2 can be classified into several genotypes or clusters [5]. Although PCV2a, PCV2b, and PCV2d are widely spread, PCV2b and PCV2d are the most commonly found in diseased pigs nowadays [6,7]. The information about PCV2 diversity in Poland is limited. Fabisiak et al. [8] showed that both PCV2a and PCV2b were present in the wild boar population in 2007, while in domestic pigs, only PCV2b was detected (Podgorska and Stadejek, unpublished, GenBank acc. EU444004-EU444010).

Vaccination is considered to be one of the most effective PCVD control strategies. Commercial vaccines against PCV2 are based on PCV2a genotype antigens, but cross-protection against PCV2b and PCV2d has been demonstrated [9,10,11,12]. Four commercial vaccines against PCV2 are available on the Polish market. Circovac (Ceva) contains inactivated PCV2a, and it is licensed for vaccination of sows and piglets. Circoflex (Boehringer Ingelheim) and Porcilis PCV (Intervet) contain recombinant PCV2 capsid protein and can be used for the immunization of sows and piglets and only piglets, respectively. Suvaxyn PCV (Zoetis) is licensed for piglets, and it is based on inactivated chimeric PCV1 virus containing the *cap* gene from PCV2a. According to all manufacturers’ instructions, it is recommended to vaccinate piglets at the age of three weeks, but the vaccination of older pigs (even at 5–6 weeks old) is sometimes practiced in the field.

It is well documented that immunization against PCV2 can limit viremia, virus shedding, and pathological lesions [13,14,15,16,17,18,19,20,21,22,23]. In effect, vaccination against PCV2 reduces the mortality rate and improves production parameters, such as average daily weight gain (ADWG) [16,17,18,19,24,25,26]. On the other hand, there have been reports on PCV2 vaccination failures that have resulted in the appearance of clinical signs of PCVD [9,27,28,29,30]. 

Porcine circovirus type 2-associated diseases can be suspected based on an assessment of clinical signs and pathological lesions, although most are nonspecific [2]. Immunohistochemistry (IHC) and hybridization *in situ* (ISH), which allow for a semi-quantitative evaluation of the presence of viral antigens or DNA in lymphoid or other tissues, as well as an evaluation of microscopic lesions used to be the golden standard of laboratory diagnosis of PCVD. The above-mentioned elements were part of the diagnostic criteria developed by Sorden in 2000 [31]. 

The common use of highly efficacious vaccines against PCV2 has nearly eliminated the occurrence of severe cases of PCVD and has increased the importance of subclinical disease. The diagnosis of subclinical disease with methods that are standard for PCVD is problematic. Quantitative or semi-quantitative PCR methods are currently used to detect and measure PCV2 loads in serum or tissues. Low Ct (cycle threshold) values or high-genome-copy equivalents (e.g., >6.0 log_10_ PCV2 genome copies/mL) detected in PCR may suggest the involvement of PCV2 in a given disease condition [2]. However, PCR-based methods and the diagnostic criteria of PCV2-related health problems are not standardized between diagnostic laboratories, and the interpretation of results is often subjective. Moreover, there is limited information about PCV2 detection rates and loads in different materials from farms using PCV2 vaccines, which would be needed to establish diagnostic benchmarks.

The aim of this study was to assess the detection rates of PCV2 in different clinical materials from pigs from farms that apply different PCV2 vaccination schemes and from non-vaccinated farms. We intended to propose PCR diagnostic criteria for the evaluation of the impact of vaccination against PCV2 on viremia and virus shedding. Additionally, a sequence analysis of PCV2 from pigs that were highly positive in the PCR was performed in order to assess the current genetic diversity of the virus in Poland.

## 2. Materials and Methods

### 2.1. Study Farms

The study was performed on 26 randomly selected commercial Polish pig farms that have different systems of production, general health statuses, hygiene levels, and vaccination protocols against PCV2. Thus, four farms in the sample do not perform any vaccination against PCV2 (NON-VAC), and 22 farms (VAC) use two different vaccination strategies: the vaccination of piglets (VAC1, 11 farms) and the vaccination of sows and their progeny (VAC2, 11 farms). On 11 farms (four NON-VAC and seven VAC farms), clinical signs resembling PCVD were observed in some pigs of different ages, but a proper laboratory investigation was not performed to confirm the cause of the disease. Detailed information about the farms is presented in Table 1.

### 2.2. Sample Collection and Processing

The samples were obtained as part of routine diagnostic or monitoring protocols, so the agreement of the local ethics committee was not required. Samples were collected from different age groups from random pigs at about 3–21 weeks of age. From each age group, from 6 to 10 blood and fecal samples were obtained. Blood was obtained through venipuncture of the *vena cava cranialis*. Fecal samples were collected by rectal swabbing of the same pigs at the time of bleeding. Additionally, one oral fluid sample was collected from each pen of pigs. In some farms, oral swabs from suckling piglets were collected using cotton swabs. In 3-week-old piglets from Farm U and in pigs older than 13 weeks from Farm P, only serum samples were collected (Table 1). Immediately after collection, the samples were chilled and transported to the laboratory of the Department of Pathology and Veterinary Diagnostics of the Faculty of Veterinary Medicine at the Warsaw University of Life Sciences. Fecal and oral fluid swabs from 3-week-old piglets were suspended in 1 mL of phosphate-buffered saline (PBS) and vortexed for five minutes, and mechanical impurities were spun down. Altogether, 1748 blood, 1717 fecal, and 387 oral fluid samples were collected. Sera, fecal swabs, and oral fluids were stored at −20 °C.

### 2.3. Real-Time PCR

Prior to DNA extraction, equal volumes of pig serum, feces, and oral fluid from 3-week-old piglets were pooled 2–6, and each pool corresponded to one pen of weaners or fatteners or one litter of suckling piglets. Total DNA was extracted from 200 µL of serum, fecal suspension, and oral fluid pools using QIAmp DNA Mini Kit or QIAmp Cador Pathogen Mini Kit (Qiagen, Hilden, Germany) according to the manufacturer’s instructions. In total, 359 pools of serum, 351 pools of feces, and 209 oral fluid samples were extracted. Extracted DNA (2.0 µL) was used to amplify by real-time PCR assay a section of the ORF2 of PCV2 and to discriminate between PCV2a and PCV2b (Table S1) [32]. As the PCV2b probe used also binds to the PCV2d genome, the PCV2b-positive results were interpreted as proof of the presence of PCV2b, PCV2d, or both (described as PCV2b/d-positive). The reaction was conducted in a Rotor Gene-Q 6000 (Qiagen) instrument under the following thermal conditions: 95 °C/2 min, followed by 40 cycles of 95 °C/5 s and 60 °C/20 s (acquiring). Samples with Ct values ≤37 were considered positive. The quantification of viral genome copies was performed using titrated plasmids containing ORF2 of PCV2 [33]. Viral titers inferred from the real-time PCR results are expressed as the log_10_ viral copy number per milliliter of pool or oral fluid (copies/mL).

### 2.4. DNA Sequence Analysis

In order to precisely determine the genotype of the detected PCV2 and to evaluate the genetic diversity of current Polish strains, DNA from the samples with the highest PCV2 loads (>6.0 log_10_ copies/mL) were subjected to PCR amplification of the ORF2 fragment (corresponding to ORF2 nucleotides 18–666) and the sequencing of the amplicons (Table S1) [34]. The sequences were aligned using a MUSCLE algorithm, and the phylogenetic tree was constructed with Geneious Tree Builder using the Geneious 10.2.6 program (Biomatters Ltd., Auckland, New Zealand).

### 2.5. Statistical Analysis

A statistical analysis was performed using GraphPad Prism 8 for Windows (GraphPad Software, San Diego, California USA, www.graphpad.com). The prevalence of PCV2 in pigs both vaccinated and non-vaccinated against PCV2 was compared using Fischer’s exact test. A comparison of log_10_ PCV2 viral loads (copies/mL) was made using the nonparametric Mann–Whitney test. A two-tailed *p*-value < 0.05 was set as the statistically significant level. In order to determine the probability of PCV2 detection in the different types of samples, age groups, and vaccination protocols used, a logistic regression analysis was performed using R software version 3.5.1 [35] and 5.1–3 *rms* package version [36].

## 3. Results

### 3.1. Detection of PCV2 in Samples from Pigs from Farms that Vaccinate (VAC) and Do Not Vaccinate (NON-VAC) against PCV2

**Figure 1:**
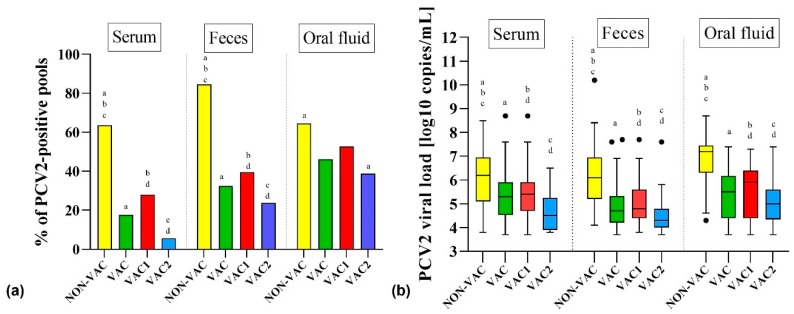
Detection of porcine circovirus type 2 (PCV2) in serum and fecal pools and in oral fluid samples from farms that do not vaccinate against PCV2 (NON-VAC) and from farms that vaccinate (VAC) using two different vaccination strategies: the vaccination of piglets (VAC1) or the vaccination of sows and their progeny (VAC2). (**a**) Percentage of PCV2-positive samples. Statistically significant differences (*p* < 0.05, Fisher’s exact test) are marked with superscripts on the tops of the bars in the chart (a, b, c, d). (**b**) Comparison of PCV2 viral loads (log_10_ copies/mL). The whisker plot uses a Tukey determination of whiskers. A statistical comparison was performed using the Mann–Whitney test. Statistically significant differences (*p* < 0.05) are marked with superscripts on the tops of the boxes in the chart (a, b, c, d).

Porcine circovirus type 2 was detected in 25 out of 26 farms (96.2%) in at least one sample. Only samples from Farm Q (VAC2) reacted negatively for PCV2 (Table 1). There were striking differences in the PCV2 detection patterns in different types of samples and between NON-VAC and VAC farms. The detection rate of PCV2 in serum pools was significantly (*p* < 0.05) higher in NON-VAC (33 out of 52, 63.5%) than in VAC farms (54 out of 307, 17.6%) (Table S2, Figure 1). Similarly, the PCV2 loads detected in serum pools from NON-VAC farms (3.8–8.5; median = 6.2 log_10_ copies/mL) were significantly (*p* < 0.05) higher than in VAC farms (3.7–8.7; median = 5.3 log_10_ copies/mL) (Table S2, Figure 1).

Porcine circovirus type 2 was more often detected in fecal pools than in serum pools of the same pigs. In NON-VAC and VAC farms, 44 out of 52 (84.6%) and 97 out of 299 (32.4%) fecal pools reacted positively for PCV2, respectively, and this difference was statistically significant (*p* < 0.05) (Table S2, Figure 1). Porcine circovirus type 2 loads in fecal pools were significantly (*p* < 0.05) lower in VAC (3.7–7.7; median = 4.7 log_10_ copies/mL) than in NON-VAC farms (4.1–10.0; median = 6.2 log_10_ copies/mL) (Table S2, Figure 1).

The results obtained from oral fluids showed that in NON-VAC farms, 20 out of 31 (64.5%) samples reacted positively for PCV2, while in VAC farms, the virus was found in 82 out of 178 (46.1%) samples (Table S2, Figure 1). Similarly to the serum and fecal pools, PCV2 loads obtained from oral fluid samples from NON-VAC farms were significantly higher (4.3–8.7; median = 7.2 log_10_ copies/mL) than in those from VAC farms (3.7–7.4; median = 5.5 log_10_ copies/mL) (Table S2, Figure 1).

### 3.2. Detection of PCV2 in Samples from Pigs from Farms Using Two Different PCV2 Vaccination Protocols

There were clear differences in PCV2 detection rates and viral loads between the samples from farms using two different vaccination protocols. Porcine circovirus type 2 was detected in 46 out of 164 (28.0%) serum pools from VAC1 farms, and its prevalence ranged from 0.0% in Farms E and G to 66.7% in Farm F (Table 1). The level of PCV2 viremia in VAC1 farms was 3.7–8.7 log_10_ copies/mL, with a median of 5.4 log_10_ copies/mL. The viremia of PCV2 in VAC2 farms was significantly (*p* < 0.05) less common, and it was found in only three farms (P, U, and X) in 8 out of 143 (5.6%) tested serum pools (Table S2, Figure 1). The level of PCV2 viremia (3.8–6.5; median = 4.5 log_10_ copies/mL) in those farms was also significantly (*p* < 0.05) lower than in VAC1 farms (Table S2, Figure 1).

The PCV2 detection rate in fecal pools was significantly (*p* < 0.05) higher in VAC1 farms (65 out of 164, 39.6%), but it ranged widely from 0.0% in Farm E to 91.7% in Farm F (Table 1). In feces from VAC2 farms, PCV2 was detected in 23.7% (32 out of 135) pools, and it was the highest in Farms U and Z (66.7% in each) (Table 1). Similarly, the level of PCV2 loads in VAC1 farms (3.8–7.7; median = 4.8 log_10_ copies/mL) was significantly (*p* < 0.05) higher than in VAC2 farms (3.7–7.6; median = 4.3 log_10_ copies/mL) (Table S2, Figure 1).

The virus was detected in 49 out of 93 (52.7%) oral fluid samples from VAC1 farms. In VAC2 farms, PCV2 was less common, and it was found in 33 out of 85 (38.8%) oral fluid samples (Table S2, Figure 1). The PCV2 viral loads in oral fluids were significantly (*p* < 0.05) lower in VAC2 farms (3.7–7.4; median = 5.0 log_10_ copies/mL) than in VAC1 farms (3.7–7.3; median = 5.9 log_10_ copies/mL) (Table S2, Figure 1).

### 3.3. Detection of PCV2 in Samples from Different Age Groups of Pigs

**Figure 2:**
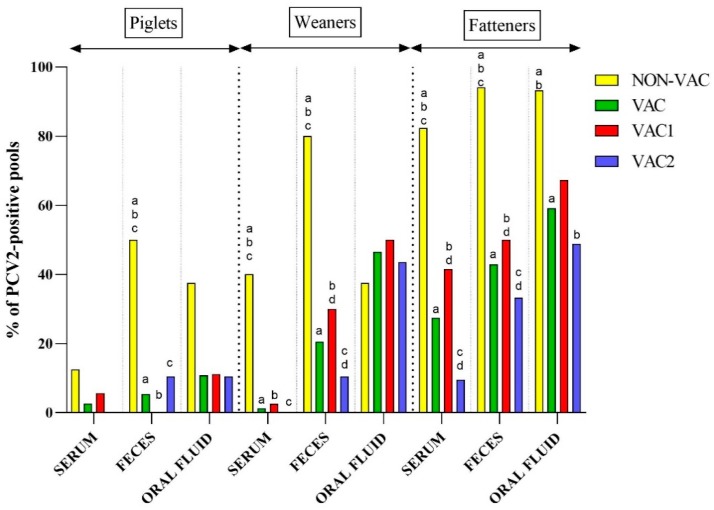
Percentage of porcine circovirus type 2 (PCV2) detection in serum and fecal pools and in oral fluid samples collected from piglets (3–4 weeks old), weaners (5–8 weeks old), and fatteners (≥9 weeks old) from farms that do not vaccinate against PCV2 (NON-VAC) and from farms that vaccinate (VAC) using two different vaccination strategies: the vaccination of piglets (VAC1) or the vaccination of sows and their progeny (VAC2). Statistically significant differences (*p* < 0.05, Fisher’s exact test) are marked with superscripts on the tops of the bars in the chart (a, b, c, d).

Suckling piglets (3–4 weeks old) were the age group with the lowest PCV2 detection rate in all kinds of diagnostic material (irrespective of the vaccination protocol). In NON-VAC farms, the virus was found in 33.3% (8 out of 24) of all tested samples (serum, feces, and oral fluids taken together) from this age group (Table S3). On the other hand, in VAC farms, the virus was found in only 6.2% (7 out of 113) of all piglet samples. In detail, PCV2 was found in a single serum pool from VAC1 farms (5.6%), two fecal pools from VAC2 farms (10.5%), and four oral fluid pools from both VAC1 and VAC2 farms (10.8%) (Figure 2, Table S3). Quantitative PCR results from all types of materials showed that the median of PCV2 viral loads was 4.5, 3.8, 4.8, and 3.8 log_10_ copies/mL in NON-VAC, VAC, VAC1, and VAC2 farms, respectively (Table S3).

**Figure 3:**
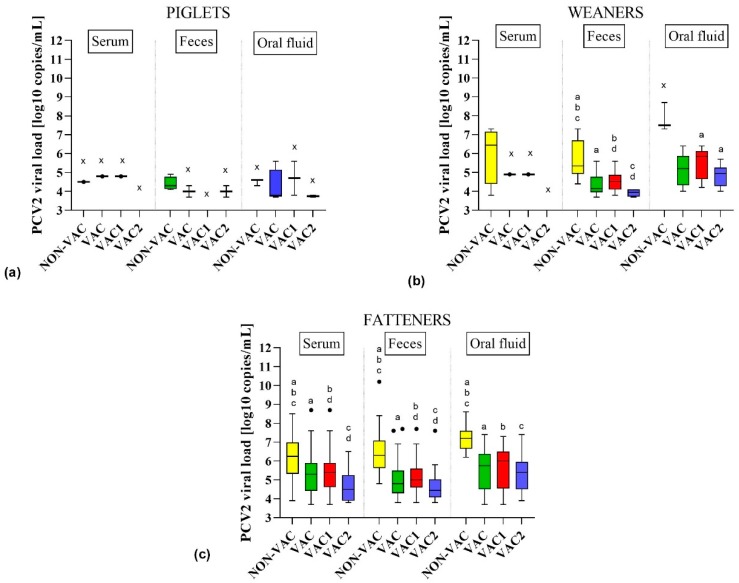
Comparison of porcine circovirus type 2 (PCV2) viral loads (log_10_ copies/mL) in serum and fecal pools and in oral fluids collected from piglets (**a**), weaners (**b**), and fatteners (**c**) from farms that do not vaccinate against PCV2 (NON-VAC) and farms that vaccinate (VAC) using two different vaccination strategies: the vaccination of piglets (VAC1) and the vaccination of sows and their progeny (VAC2). The whisker plots use a Tukey modification of whiskers. A statistical comparison was performed using the Mann–Whitney test. Statistically significant differences (*p* < 0.05) are marked with superscripts on the tops of the boxes in the chart (a, b, c, d), and “x” indicates diagnostic material that was not taken into account in the statistical comparison (too small a number of PCV2-positive samples).

Porcine circovirus type 2 DNA was more frequently found in weaners (5–8 weeks old) than in piglets. Similarly to piglets, PCV2 prevalence was significantly higher (*p* < 0.05) in NON-VAC farms (53.6% of weaner samples positive) than in VAC farms (18.1% of all weaner samples positive) (Table S3). The PCV2 viral loads detected in weaners from NON-VAC farms (3.8–8.7; median = 6.2 log_10_ copies/mL) were significantly higher than in those from VAC farms (3.7–6.4; median = 4.8 log_10_ copies/mL) (Table S3). In NON-VAC farms, PCV2 was the most frequent in fecal pools (8 out of 10, 80.0%), and the log_10_ PCV2 load ranged from 4.4 to 7.3 log_10_ copies/mL with a median of 5.4 log_10_ copies/mL (Table S3, Figure 2 and Figure 3). In VAC farms, the highest PCV2 prevalence and viral loads (20 out of 43, 46.5%, 4.0–6.4; median = 5.2 log_10_ copies/mL,) were observed in oral fluid samples from weaners (Table S3, Figure 2 and Figure 3). No statistical differences (*p* > 0.05) were found in terms of virus prevalence (23.0% vs 14.1%) and log_10_ viral loads (median of 4.8 vs 4.4 log_10_ copies/mL) between VAC1 and VAC2 farms (Table S3).

**Figure 4:**
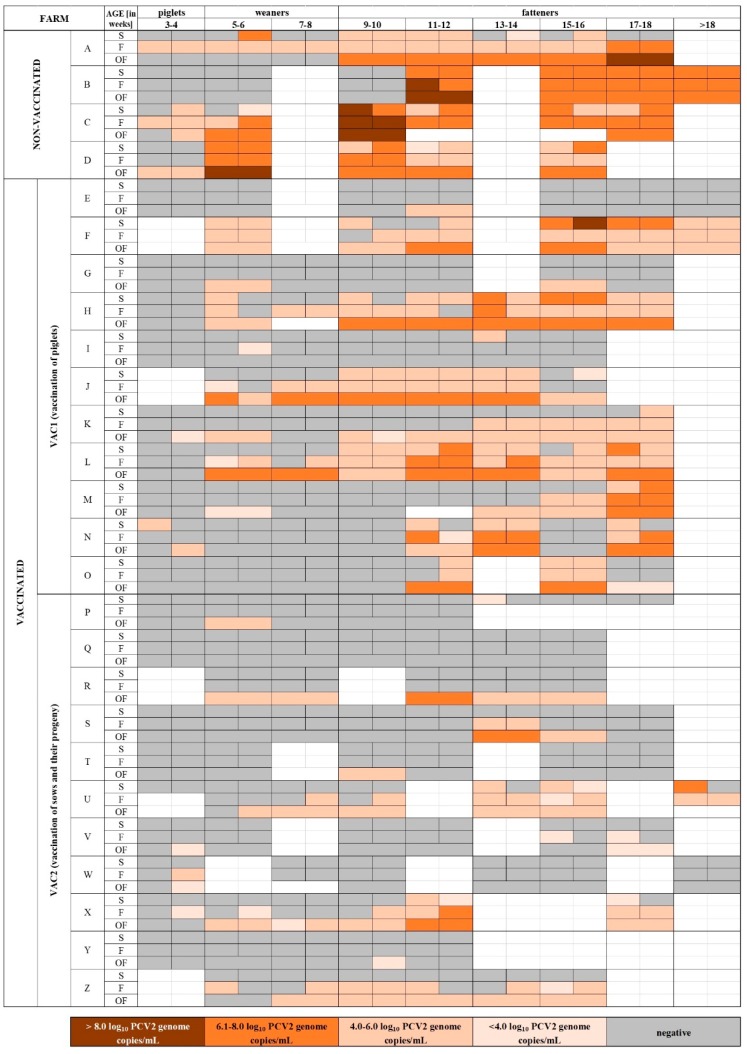
Detection of porcine circovirus type 2 (PCV2) in different materials (serum (S), feces (F), oral fluids (OFs)), age groups (piglets, weaners, and fatteners), and farms with different vaccination protocols against PCV2 (VAC1: piglet vaccination; VAC2: vaccination of sows and their progeny; NON-VAC: no vaccination). Positive pools are marked with orange cells. The negative pools are marked with gray cells. The white cells correspond to pools that were not collected. Positive samples were divided into four groups based on log_10_ PCV2 copy equivalents/mL of pool (>8.0, 6.1–8.0, 4.0–6.0, and <4.0 log_10_ copies/mL).

Fatteners (≥9 weeks old) were the group where PCV2 was the most common. Similarly to the younger pigs, PCV2 was significantly (*p* < 0.05) more frequent in NON-VAC farms (89.2% of fatteners sample positive) than in VAC farms (40.0% of fatteners sample positive) (Table S3). In addition, the PCV2 load was significantly higher in NON-VAC (3.9–10.0; median = 6.5 log_10_ copies/mL) than in VAC farms (3.7–8.7; median = 5.2 log_10_ copies/mL). More than 90.0% of fecal pools and oral fluid samples from NON-VAC fatteners were PCV2-positive (Table S3, Figure 2). In one fecal pool from Farm C, an amount of virus as high as >10.0 log_10_ copies/mL was detected (Figure 4). The virus was found in 50.2% of fattener samples from VAC1 farms and in only 26.8% of fattener samples from VAC2 farms, which was significantly different (*p* < 0.05) (Table S3). The PCV2 load in VAC1 farms (3.7–8.7; median = 5.3 log_10_ copies/mL) was significantly higher than in VAC2 farms (3.8–7.6; median = 4.8 log_10_ copies/mL) (Table S3).

### 3.4. Probability of PCV2 Detection in Different Types of Samples, Age Groups, and Vaccination Protocols

The detection of PCV2 was the most frequent in oral fluids, fatteners, and NON-VAC farms. Therefore, these factors were used as the reference levels in the logistic regression model to determine the probability of PCV2 detection in variables including different age groups (piglets, weaners, and fatteners), diagnostic materials (serum, feces, and oral fluids), and statuses of vaccination against PCV2 (NON-VAC, VAC1, and VAC2 farms). In all of the examined farms, the probability of PCV2 detection decreased by 80.8% and 51.5% in serum and feces pools compared to oral fluids, respectively (Table S5). The probability of PCV2 detection decreased by 93.4% and 74.2% in piglets and weaners, respectively, compared to fattener samples. NON-VAC farms were characterized by the highest probability of PCV2 detection compared to VAC1 and VAC2 farms (78.8% and 92.1% decrease, respectively) (Table S5).

### 3.5. Genotyping of PCV2

In this study, a real-time PCR assay allowed for discrimination between PCV2a and PCV2b/d. The real-time PCR results revealed that the most common genotypes were PCV2b/PCV2d, which were identified in 15 out of 25 positive farms (66.7%). In nine farms (36.0%), PCV2a was found, and in three of them (12.5%; farms M, O, and Y), it was the only genotype detected (Table 1). The coexistence of PCV2a and PCV2b/PCV2d was detected in six farms (25.0%) (Table 1).

In NON-VAC farms, PCV2b/PCV2d were present in all 98 PCV2-positive samples. In two of them, coexistence with PCV2a was detected. In VAC farms, PCV2b/PCV2d were also the most common, and they were detected in 180 out of 233 (77.3%) samples. Singular infection with PCV2a was found in 44 (18.9%) of the samples from VAC farms. The coexistence of PCV2a and PCV2b/PCV2d was found in nine (3.9%) samples from VAC farms.

**Figure 5:**
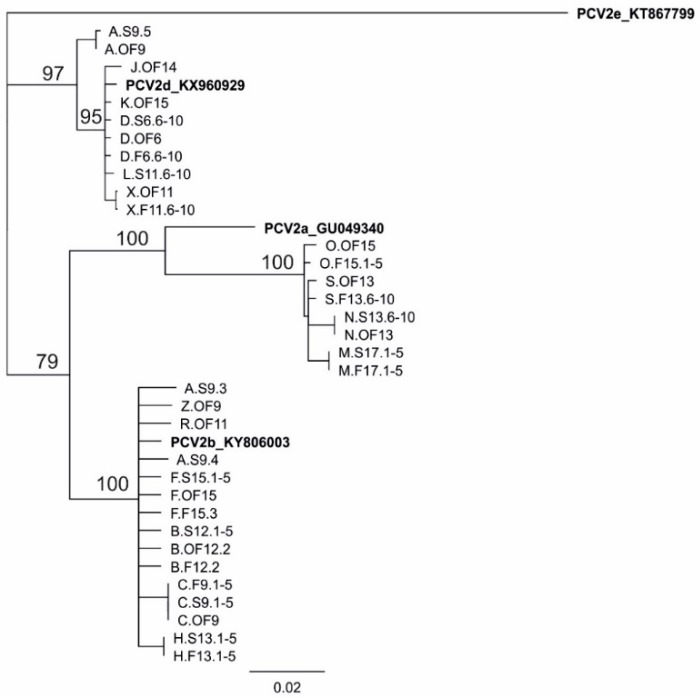
Phylogenetic tree constructed of partial ORF2 sequences of porcine circovirus type 2 (PCV2) obtained for the present study from serum (S), feces (F), or oral fluids (OFs). Reference sequences of PCV2a, PCV2b, PCV2d, and PCV2e (used as an outgroup) were obtained from GenBank. The sequences were aligned with MUSCLE, and the tree was constructed with Geneious Tree Builder using the Neighbor-Joining method with 100 replicates (using Geneious 10.2.6 (Biomatters Ltd.)). The scale bar represents the number of substitutions per site. The numbers at nodes indicate consensus support (%). The sequences obtained in this study are available in GenBank under accession numbers MN593265–MN593297.

The DNA sequencing of ORF2 fragments from samples containing >6.0 log_10_ copies/mL provided 11 sequences from serum, 9 sequences from feces, and 13 sequences from oral fluids from 16 farms (Figure 5). In addition, eight (24.2%), 15 (45.5%), and 10 (30.3%) out of 33 obtained sequences belonged to PCV2a, PCV2b, and PCV2d genotypes, respectively. Overall, 4 (25.0%), 7 (43.8%), and 6 (37.5%) out of 16 examined sequences had PCV2a, PCV2b, and PCV2d, respectively. A sequence analysis of PCV2 from a serum pool from 9-week-old pigs from Farm A showed many ambiguous nucleotide positions, indicating infection with multiple PCV2 genotypes. Indeed, the amplification and sequencing of individual serum samples from this pool showed the presence of PCV2d and two different strains of PCV2b (Figure 5).

## 4. Discussion

Porcine circovirus type 2 is a globally distributed pathogen that causes huge economic losses in the swine industry [3,4]. In 2006, the first vaccine against PCV2 was introduced in the USA [37]. Subsequently, several more products were put on the global market, and today vaccines against PCV2 are considered to be one of the most efficacious vaccines for pigs ever developed. Numerous studies on PCV2 vaccine performance have been conducted, providing strong evidence of their very high efficacy in field and experimental conditions [13,14,15,16,17,18,19,20,21,22,23,25]. However, despite the continuous use of PCV2 vaccines, the virus tends to persist in immunized populations and causes subclinical infections, sometimes confusing the differential diagnosis of disease conditions with clinical signs resembling PCVD.

In the view of increasing importance of subclinical PCV2 infections and common use of vaccination, traditional tools of PCVD diagnosis are becoming less useful and limited. Instead, quantitative or semi-quantitative real-time PCR methods are often used to detect PCV2 and to predict its role in pig health. It is assumed that vaccination limits the viremia and shedding of PCV2, but it is unclear what virus levels, e.g., in serum, are acceptable or alarming (suggestive of vaccination failure). Differences in vaccination protocols, vaccination errors, off-label use, and environmental variables may have an impact on the apparent PCV2 vaccination efficacy, which is not linked to the performance of a given product. Additionally, a limited interest in PCV2 surveillance in vaccinated farms has generated a serious gap in our knowledge on PCV2 ecology under the pressure of vaccine stimulated population immunity. Detailed studies of cross-sectional samples from farms with different vaccination statuses and clinical situations are scarce. Thus, in our study, we intended to assess PCV2 detection rates in different diagnostic materials from farms with varied vaccination statuses in order to provide some indications of what could or should be expected from a vaccination protocol in terms of real-time PCR surveillance results.

The study was performed on serum, feces, and oral fluid samples from 22 farms where PCV2 vaccination programs are in place and from 4 farms that do not vaccinate. In general, our findings support earlier observations that vaccination against PCV2 can significantly limit viremia and viral shedding in feces [13,14,15,16,17,18,19,20,21,22,23,26,38] as well as viral presence in oral fluids [34].

The PCV2 viremia was more frequently detected in NON-VAC farms than in VAC farms (Table S2, Figure 1). Additionally, we observed a significant (*p* < 0.05) reduction in viremia levels in VAC farms compared to NON-VAC farms (Table S2, Figure 1). The vaccination significantly (*p* < 0.05) limited PCV2 shedding in feces (Table S2, Figure 1). What is noteworthy, even non-viremic pigs from Farms R, S, V, W, and Z shed PCV2 in feces (Table 1). The origin of PCV2 in fecal samples, especially from non-viremic pigs, is difficult to explain. In PCV2 enteric disease (PCV2-ED), moderate-to-high amounts of the virus can be detected in Payer’s patches or intestinal mucosa [2]. Porcine circovirus type 2 can also infect the intestinal porcine epithelial cell line (IPEC-J2), which is used as a model for intestinal PCV2 pathogenesis [39]. Thus, the biology of the virus in the intestinal tract needs to be explained, as it may be crucial for future efforts of PCV2 elimination from farms.

Oral fluids are becoming an alternative sample material for PCV2 detection because of their welfare and economic bonuses [40,41,42]. However, the interpretation of PCV2 presence in this material is more difficult than in the case of serum or even feces, as it can originate from an environment in which immune pigs free from PCV2 infection live. Thus, in theory, the results of oral fluid testing may better reflect the level of hygiene in a pen than the infectious status of the sampled pig population. Indeed, our study did not show any significant differences between PCV2 prevalence in oral fluids from NON-VAC and VAC farms. However, a significant reduction (*p* < 0.05) of viral loads detected in oral fluids was found, which proved that the vaccination limits the level of environmental contamination with PCV2 (Figure 1).

The effect of sow and/or piglet vaccination against PCV2 was evaluated by Fraile et al. [43]. In that study, earlier seroconversion, a lower proportion of PCR-positive pigs, and a significant improvement in production parameters were observed in piglets vaccinated at four weeks of age in comparison to non-vaccinated groups [43]. On the other hand, the authors emphasized that the vaccination of both sows and piglets further reduced PCV2 prevalence and resulted in better production parameters. In the present study, we found significant (*p* < 0.05) differences in the incidence of PCV2 viremia in farms using different protocols of vaccination (Table S2, Figure 1). We also observed a significant (*p* < 0.05) reduction in PCV2 loads in VAC1 and VAC2 farms (Table S2, Figure 1). Similar differences were evident for fecal and oral fluid samples (Table S2, Figure 1). The majority of VAC2 farms apply PCV2 vaccination in sows and 6-week-old pigs, which has been suggested as being the most effective protocol [34,44]. In addition, in the VAC2 group, in Farms Q, T, and Y, no PCV2 viremia or shedding with feces was detected. On the other hand, in the VAC1 group, there were two farms (E and G, Table 1) that were free from PCV2 viremia and shedding in feces. Thus, it is clear that the vaccination of piglets alone can be as effective as the vaccination of sows and their progeny. However, it has to be kept in mind that relatively few farms from both groups were tested, and they varied widely in size, management practices, the level of biosecurity, and hygiene levels. This made an ultimate conclusion on the advantage of one vaccination scheme over the other difficult.

Two VAC1 farms and eight VAC2 farms were found to be free from PCV2 viremia (Table 1). This apparently inferior effect of the VAC1 protocol on PCV2 viremia could be explained by a detailed analysis of the PCR results of the individual farms. In four VAC1 farms (F, H, J, and L, Table 1), a very high prevalence of PCV2 in serum, feces, and oral fluids was detected. The PCV2 viral loads in sera from Farms F, H, and L (Table 1) were comparable (*p* > 0.05) to those of NON-VAC farms. Only in Farm J a significant reduction (*p* < 0.05) in viremia was observed. This suggests serious problems with the efficacy of the vaccination protocol in Farms F, H, and L, as measured by the level of viremia and shedding with feces. The elucidation of these observations was out of the scope of this study, but it has to be underlined that in many instances, vaccines (not just those for PCV2) are used off-label (e.g., a partial dose application), stored in inadequate conditions, or applied to sick, immunocompromised animals. These conditions could certainly lead to the limited efficacy of a PCV2 vaccination protocol in a given situation. Interestingly, when the farms F, H, J, and L were excluded from the comparison, the prevalence of PCV2 and viral loads in VAC1 and VAC2 farms was found to be similar (*p* > 0.05) (Table S4). This clearly indicated that the vaccination of piglets or sows and piglets can be equally efficient in terms of limiting viremia and virus shedding, and other factors may be more important for vaccination efficacy than the protocol itself.

Porcine circovirus type 2 was generally the easiest to detect in finishers (>9 weeks of age), regardless of the vaccination status of pigs, but prevalence and viral loads were significantly (*p* < 0.05) lower in VAC than in NON-VAC farms (Figure 2 and Figure 3, Table S3). This indicated that vaccination against PCV2 protected not only against clinical disease in fatteners, but also provided long-lasting limiting effects on virus circulation.

Our analysis showed very different patterns of PCV2 detection in individual farms. In VAC2 Farm Q, PCV2 was not detected in any of the samples. In VAC1 Farms E and G and in VAC2 Farms T and Y, PCV2 was detected in one or two oral fluid samples, despite the lack of viremia and shedding in feces. The lack of PCV2 viremia and shedding in feces may indicate that the farms were approaching PCV2-free status at the time of sampling. Interestingly, the veterinary consultants dealing with Farms Q and G reported observations of PCVD-like clinical signs and expressed concerns regarding PCV2 vaccination efficacy (Table 1). This underlines the importance of careful diagnostic investigations in cases with suspected PCV2 vaccination failure.

The practically relevant question is whether PCV2 can be eliminated from a farm. In farms where good vaccination practices are in place, a declining trend in PCV2 prevalence has been described [45,46], but PCV2 eradication using vaccination alone is questionable [37,47]. Feng et al. [47] have reported that stopping vaccinations resulted in PCV2 reinfection, probably from the contaminated environment. The single positive results from oral fluid testing in otherwise PCV2-free Farms E, G, T, and Y clearly indicate some level of contamination with PCV2, which could pose a significant risk for naïve pigs in the case of a vaccination protocol being abandoned.

Before 2000, PCV2a was said to be the predominant PCV2 genotype [48]. In 2005, a global shift toward PCV2b [49] and then another shift to PCV2d were observed [6]. Considering that vaccination against PCV2 is common, sequence analyses and genotyping are more difficult tasks than they were previously. In our study, samples with viral loads >6.0 log_10_ copies/mL, which are appropriate for ORF2 sequencing, were found about three times more often in NON-VAC farms than in VAC farms (Figure 4). Thus, genotype-specific PCR methods would certainly be helpful for a detailed analysis of PCV2 genotype prevalence. Our results clearly indicate that all three major PCV2 genotypes (PCV2a, PCV2b, and PCV2d) infect pigs in Poland (Table 1, Figure 5). The detection of PCV2a in commercial Polish pig farms was surprising considering that a previous report about PCV2 genetic diversity in Poland described only PCV2a in wild boars [8].

According to previous studies [10,11,12,23], the genetic diversity of PCV2 should not have any impact on the efficacy of PCV2a-based vaccines. In our research, in the VAC group, there were no statistical differences in terms of PCV2 loads between PCV2a-positive farms, PCV2b/d-positive farms, and farms with PCV2a and PCV2b/d coexistence (Table 1). However, virus prevalence was significantly the highest (*p* < 0.05) in farms with PCV2b/d infection (36.2%). In the farms suspected of PCV2 vaccination failures, PCV2b (Farm F and H) and PCV2d (Farm J) as well as PCV2a and PCV2d (Farm L) were identified on the basis of the real-time PCR and sequence analysis, which indicates that other factors played a role in limiting the vaccine’s impact on viremia (Table 1, Figure 5).

Porcine circovirus type 2 diagnosis in pig herds can be a challenge nowadays. Our observations and the results of the logistic regression model showed that the probability of PCV2 detection was the highest in oral fluids and fatteners (Table S5). Thus, oral fluids seem to be a reliable material for PCV2 detection on an environmental level, especially in vaccinated farms. In order to determine the effectiveness of vaccinations, the testing of serum samples can be recommended. The median of the PCV2 viral loads in pooled sera >5.0 log_10_ copies/mL can be considered an indication of PCVD in some animals in the sampled population. The detection of viral levels comparable to those in farms that do not vaccinate (i.e., >6.0 log_10_ copies/mL) should be considered to be a very strong sign of PCVD being a serious problem in the sampled population. Obviously, a revision of the vaccination protocol should be considered. The elimination of PCV2 viremia and subclinical disease is certainly possible through systematic vaccination and may lead to more efficient pig production.

## 5. Conclusions

Despite vaccination against PCV2, pig farms can exhibit very different patterns of PCV2 viremia and shedding in feces and in terms of its presence in oral fluids. Vaccination can successfully reduce or eliminate PCV2 prevalence and viral loads, but many factors, such as hygiene level, biosecurity, farm management, and errors related to vaccine handling and administration, may affect this outcome. Oral fluids testing with PCR can be recommended to monitor PCV2 elimination from farms, but the examination of viremia seems to be the key element in the diagnosis of PCV2-related problems in vaccinated farms. The detection of high viral loads in pools of serum (i.e., >6.0 log_10_ copies/mL) in vaccinated populations should be considered unacceptable, and corrective measures should be developed and applied.

## Figures and Tables

**Table 1:** Farm characteristics and summary results of real-time PCR for porcine circovirus type 2 (PCV2) in serum (S) and fecal (F) pools and oral fluids (OFs). Diagnostic materials with PCV2a and PCV2b/d coexistence on the herd (*) or pen level (**) are marked: “neg” indicates a negative result (cycle threshold (Ct) >37). Health status was reported by veterinary practitioners supervising the farms based on subjective assessments. PCVD: PCV2-associated disease.

Vaccination Against PCV2 Protocol		Farm ID	Health Status and Clinical Signs	% of PCV2-Positive Pools			PCV2 Viral Load			% of PCV2a-Positive Pools			% of PCV2b/d-Positive Pools		
				(Positive/All Tested)			(Minimum–Maximum, Median) (log_10_ copies/mL)			(Positive/PCV2-Positive)			(Positive/PCV2-Positive)		
				S	F	OF	S	F	OF	S	F	OF	S	F	OF
**NON-VACCINATED**		**A**	poor	43.8	100	45.5	3.9-6.7,	4.1–7.1,	4.3–5.9,	neg	7.3	20.0	100	100	100
			PCVD	(7/16)	(16/16) **	(5/11) **	5.3	5.2	5.4		(1/16)	(1/5)	(7/7)	(16/16)	(5/5)
		B	poor	57.1	57.1	50.0	6.2–7.6,	6.3–8.1,	5.1–8.2,	neg	neg	neg	100	100	100
			PCVD	(8/14)	(8/14)	(4/8)	6.9	6.9	6.5				(8/8)	(8/8)	(4/4)
		C	poor	83.3	100	83.3	3.8–8.5,	4.4–10,	7.1–8.3,	neg	neg	neg	100	100	100
			PCVD	(10/12)	(12/12)	(5/6)	6.2	6.8	7.2				(10/10)	(12/12)	(5/5)
		D	poor	80.0	80.0	100	3.9–7.4,	4.8–7.3,	4.3–8.6,	neg	neg	neg	100	100	100
			PCVD	(8/10)	(8/10)	(6/6)	6.0	6.1	7.2				(8/8)	(8/8)	(6/6)
**VACCINATED**	**VAC1** (vaccination of piglets)	E	very good	neg	neg	12.5 (1/8)	neg	neg	5.1	neg	neg	neg	neg	neg	100 (1/1)
		F	average	66.7	91.7	100	4.2–8.7,	4.1–5.7,	4.7–6.8,	neg	neg	neg	100 (8/8)	100 (11/11)	100 (6/6)
			PCVD	(8/12)	(11/12)	(6/6)	6.0	5.1	5.9						
		G	average	neg	neg	22.2	neg	neg	4.2–4.4,	neg	neg	neg	neg	neg	100
			PCVD			(2/9)			4.3						(2/2)
		H	average	62.5	75.0	75.0	4.3–7.6,	4.0–6.3,	5.8–7.0,	neg	neg	neg	100	100	100
			PCVD	(10/16)	(12/16)	(6/8)	5.3	4.6	6.4				(10/10)	(12/12)	(6/6)
		I	good	7.1	7.1	neg	4.2	4.1	neg	neg	100	neg	100	neg	neg
				(1/14)	(1/14)						(1/1)		(1/1)		
		J	average	58.3	75.0	100	3.7–5.6,	3.9–5.6,	5.5–6.8,	neg	neg	neg	100	100	100
				(7/12)	(9/12)	(7/7)	5.2	4.8	6.1				(7/7)	(9/9)	(7/7)
		K	average	6.3	37.5	80.0	5.2	4.0–5.0,	3.8–6.0,	neg	neg	25.0	100	100	87.5
				(1/16)	(6/16)	(8/10) **		4.5	4.8			(2/8)	(1/1)	(6/6)	(7/8)
		L	average	56.3	81.3	80.0	3.9–7.4,	3.8–6.3,	4.3–7.0,	44.4	61.5	12.5	77.8	76.9	100
				(9/16) **	(13/16) **	(8/10) **	5.8	4.9	6.3	(4/9)	(8/13)	(1/8)	(7/9)	(10/13)	(8/8)
		M	average	12.5	25.0	50.0	5.9–6.0,	4.3–6.1,	4.2-7.3,	100	100	100	neg	neg	neg
				(2/16)	(4/16)	(4/8)	5.9	5.3	5.1	(2/2)	(4/4)	(4/4)			
		N	average	31.3	37.5	44.4	4.6–5.8,	3.8–7.7,	4.4–6.3,	80.0	100	75.0	20	neg	25.0
				(5/16) *	(6/16)	(4/9) *	4.8	6.3	5.9	(4/5)	(6/6)	(3/4)	(1/5)		(1/4)
		O	average	18.8	18.8	33.3	4.5–5.3,	5.2–5.4,	3.7–7.3,	100	100	100	neg	neg	neg
				(3/16)	(3/16)	(3/9)	4.7	5.3	6.1	(3/3)	(3/3)	(3/3)			
	**VAC2** (vaccination of sows and their progeny)	P	very good	6.3	neg	16.7	3.8	neg	neg	neg	neg	100	100	neg	neg
				(1/16)		(1/6)						(1/1)	(1/1)		
		Q	average	neg	neg	neg	neg	neg	neg	neg	neg	neg	neg	neg	neg
			PCVD												
		R	average	neg	40.0	100	neg	3.8–4.8,	5.0–6.1,	neg	neg	neg	neg	100	100
			PCVD		(4/10)	(5/5)		4.2	5.2					(4/4)	(5/5)
		S	average	neg	12.5	22.2	neg	5.7–5.8,	4.4–7.4,	neg	100	50	neg	neg	50
											(2/2)	(1/2)			(1/2)
					(2/16)	(2/9) *		5.8	5.9						
		T	average	neg	neg	14.3	neg	neg	4.5	neg	neg	neg	neg	neg	100
						(1/7)									(1/1)
		U	average	28.6	66.7	83.3	3.9–6.5,	3.8–5.0,	4.3–4.9,	neg	neg	neg	100	100	100
				(4/14)	(8/12)	(5/6)	4.8	4.1	4.5				(4/4)	(8/8)	(5/5)
		V	average	neg	16.7	28.6	neg	3.8–3.9,	3.7–3.9,	neg	neg	neg	neg	100	100 (2/2)
					(2/12)	(2/7)		3.9	3.8						
		W	average	neg	8.3	16.7	neg	4.3	3.8	neg	neg	neg	neg	100	100
					(1/12)	(1/6)								(1/1)	(1/1)
		X	average	20.0	46.7	64.3	3.9–5.1,	3.7–7.6,	4.0–7.1,	neg	neg	neg	100	100	100
			PCVD	(3/15)	(7/15)	(9/14)	4.8	5.1	5.7				(3/3)	(7/7)	(9/9)
		Y	average	neg	neg	11.1	neg	neg	3.9	neg	neg	100	neg	neg	neg
						(1/11)						(1/1)			
		Z	average	neg	66.7	85.7	neg	3.8–4.8,	4.3–5.9,	neg	neg	neg	neg	100	100
														(8/8)	(6/6)
			PCVD		(8/12)	(6/7)		4.4	5.4						

## Supplementary Materials

The following are available online at https://www.mdpi.com/1999-4915/11/12/1135/s1, Table S1: Primers and probes used for PCV2 detection, differentiation, and sequencing; Table S2: Summary of the real-time PCR results for porcine circovirus type 2 (PCV2) in the serum pools, fecal pools, and oral fluid samples obtained from nonvaccinated and vaccinated farms; Table S3: Summary of the real-time PCR results for porcine circovirus type 2 (PCV2) in the serum pools, fecal pools, and oral fluid samples collected from different age groups; Table S4: Prevalence and viral loads of PCV2 after the exclusion of farms suspected of vaccination against PCV2 failures; Table S5: Boxplots of PCV2 detection in variables (diagnostic materials and age group) using the logistic regression model.
