# Supplementary material for: Real-Time PCR Detection Patterns of Porcine Circovirus Type 2 (PCV2) in Polish Farms with Different Statuses of Vaccination against PCV2
Source: Viruses. 2019 Dec 8;11(12):1135. doi: 10.3390/v11121135 (PMC6949947; doi:10.3390/v11121135)
Supplement: Supplementary file 1 [file viruses-11-01135-s001.pdf]

Table S4. Comparison of porcine circovirus type 2 (PCV2) vaccination status: farms non-vaccinated against PCV2 (NON-VAC) and vaccinated farms (VAC) and vaccination protocols: vaccination of piglets (VAC1) or vaccination of sows and their progeny (VAC2). Viral loads based on real-time PCR were expressed as log_{10} viral copies number per milliliter of a pool or oral fluid (copies/mL). Statistical comparisons of percentages (Fisher's exact test) and viral loads (Mann-Whitney test) of total PCV2-positive samples for farms with different vaccination protocol were performed. Statistical significant differences ($p<0.05$) are marked with superscripts (a, b, c and A, B, C).
The table exclude farms with clinical sings resembling PCV2-associated diseases (PCVD) and high detection rate of PCV2 in serum, feces and oral fluids.

**% of PCV2-positive samples and PCV2 log_{10} viral load (minimum – maximum; median [copies/mL])**					
**Diagnostic material**		NON-VAC	VAC	VAC1	VAC2
**serum**	%	63.5% (33/52) [a]	5.6% (20/251) [a]	11.1% (12/108)	5.6% (8/143)
	PCV2 viral load	3.8-8.5 6.2 [A]	3.8-6.5 4.8 [A]	4.2-6.0 5.0	3.8-6.5 4.5
**feces**	%	84.6% (44/52) [b]	21.4% (52/243) [b]	18.5% (20/108)	23.7% (32/135)
	PCV2 viral load	4.1-10.0 6.2 [B]	3.7-7.7 4.5 [B]	3.8-7.7 4.9 [D]	3.7-7.6 4.3 [D]
**oral fluid**	%	64.5% (20/31) [c]	37.4% (55/147) [c]	35.5% (22/62)	38.8% (33/85)
	PCV2 viral load	4.3-8.7 7.2 [C]	3.7-7.4 5.1 [C]	3.7-7.3 5.1	3.7-7.4 5.0
